# Pregnancy in GNE myopathy patients: a nationwide repository survey in Japan

**DOI:** 10.1186/s13023-020-01487-5

**Published:** 2020-09-11

**Authors:** Wakako Yoshioka, Naoyuki Miyasaka, Ryo Okubo, Reiko Shimizu, Yuji Takahashi, Yuriko Oda, Ichizo Nishino, Harumasa Nakamura, Madoka Mori-Yoshimura

**Affiliations:** 1 Department of Neuromuscular Research, National Institute of Neuroscience, National Center of Neurology and Psychiatry (NCNP), 4-1-1 Ogawa-higashi-cho, Kodaira, Tokyo, Japan; 2 Institute of Medical Genetics, Tokyo Women’s Medical University, 8-1 Kawada-cho, Shinjuku-ku, Tokyo, Japan; 3 Comprehensive Reproductive Medicine, Graduate School of Medical and Dental Sciences (Medicine), Tokyo Medical and Dental University, 1-5-45 Yushima, Bunkyo-ku, Tokyo, Japan; 4 Department of Clinical Epidemiology, Translational Medical Center, NCNP, Tokyo, Japan; 5 Department of Clinical Research Promotion, Translational Medical Center, NCNP, Tokyo, Japan; 6 Department of Neurology, National Center Hospital, National Center of Neurology and Psychiatry (NCNP), 4-1-1 Ogawa-Higashi, Kodaira, Tokyo, 187-8502 Japan; 7 Patient Association for Distal Myopathies, 2-2-15 Hamamatsucho, Minato-ku, Tokyo, Japan

**Keywords:** GNE myopathy, Pregnancy, Delivery, Threatened abortion, Disease progression

## Abstract

**Background:**

GNE myopathy is an autosomal recessive adult-onset distal myopathy. While a few case reports have described the progression of GNE myopathy during pregnancy, to our knowledge, none have examined disease progression after delivery or obstetric complications.

**Objective:**

This study aimed to reveal maternal complications, newborn complications, and the impact of pregnancy on disease progression in GNE myopathy patients.

**Methods:**

We conducted a questionnaire survey on pregnancy, delivery, and newborns involving female GNE myopathy patients who are currently registered in a national registry in Japan.

**Results:**

The response rate for the questionnaire survey was 60.0% (72/120). Of the 72 respondents, 44 (61.1%) had pregnancy experience (average, 1.8 pregnancies; 53 pregnancies before onset and 28 after onset). The incidence of threatened abortion was 26.9% among post-onset pregnancies, which was higher compared to those of the general Japanese population (*p* = 0.03). No other maternal or infant complications were commonly observed. Over 80% were unaware of changes in disease progression during pregnancy (mean age, 32.8 ± 3.5 years) or after delivery (32.9 ± 3.8 years), while 19.0% experienced disease exacerbation within a year after delivery (30.0 ± 1.0 years). Six patients developed myopathy within a year after delivery (29.7 ± 4.6 years), while none developed myopathy during pregnancy.

**Conclusions:**

There were no serious maternal or newborn complications, and subjective progression did not differ during or after delivery in the majority of GNE myopathy patients. However, our findings suggest the importance of considering the possibility of threatened abortion and disease progression after delivery.

## Background

GNE myopathy is a rare autosomal recessive adult-onset distal myopathy caused by biallelic pathogenic variants in the *GNE* gene, which encodes UDP-N-acetylglucosamine (UDP-GlcNAc) 2-epimerase/N-acetylmannosamine (ManNAc) kinase, a protein with key enzymatic activities in sialic acid biosynthesis [1–5]. Proof of concept of sialic acid supplementation therapy has been demonstrated in mouse models [6, 7]. However, a Phase 3 clinical trial found that aceneuramic acid extended-release tablets were not effective in treating GNE myopathy [8]. Clinical trials involving ManNAc, a precursor in the sialic acid biosynthetic pathway, demonstrated that it is safe and well tolerated [9], and a Phase 3 trial will begin in the near future. Notwithstanding, no definitive therapy for GNE myopathy currently exists.

The onset of GNE myopathy typically occurs in the 20s–40s, which corresponds to the reproductive age of women [10]. A number of reports have touched on the impact of the disease on pregnancy. For instance, in a study in Thailand, 3 GNE myopathy patients developed rapid deterioration of muscle weakness during pregnancy [11]. Similarly, the development of myopathy was noted in an Egyptian patient [12] and a Korean patient [13] during their first pregnancies. While these studies suggest the possibility that pregnancy may trigger disease onset, there have been no reports on maternal and newborn complications and disease progression after delivery. In theory, loss of sialic acid due to breastfeeding could exacerbate disease progression. Concentrations of sialic acid in breast milk are reportedly 1240 ± 229, 881 ± 273, and 505 ± 251 mg/kg in colostrum, transitional, and mature milk, respectively [14]. These collectively correspond to a maximum deficiency of 1.7 g sialic acid/day in the mother [15].

We have collected data on the course of pregnancy and delivery outcomes of female GNE myopathy patients in order to identify issues and develop recommendations to help obstetricians guide their patients, as well as to gain a deeper understanding of pregnant GNE myopathy patients and those who wish to become pregnant. The present study provides the first collective set of data on pregnancy and delivery outcomes of GNE myopathy patients.

## Materials and methods

### Registration

A national registry for neuromuscular diseases in Japan (Remudy; http://www.remudy.jp/) was developed in 2009 and supported by Intramural Research Grants (26–7) for Neurological and Psychiatric Disorders from the National Center of Neurology and Psychiatry (NCNP). Details regarding the registry have been described previously [10, 16]. The diagnosis of registered GNE myopathy patients was confirmed genetically or pathologically.

### Ethics approval and patient consent

All patients provided informed consent to share data collected in the Remudy database upon request, and the registration process was approved by Medical Ethics Committee of the NCNP (A2011–079). The present study was also approved by the same committee (A2018–105), and the objective, design, risks, and benefits of the study were explained to all participants. Consent was implied when patients completed and returned their questionnaires.

### Participants and questionnaire survey

A questionnaire with a linkable anonymized ID was distributed to 122 female GNE myopathy patients who were registered in the Remudy database as of April 2019. The questionnaire was mailed to the patients, and those who responded did so by postal mail or e-mail (PDF files) via the Remudy homepage on an anonymous and voluntary basis. Reminders were sent to those who had not responded. The Patient Association of Distal Myopathies (PADM) also emailed their patients to request cooperation with the survey.

The questionnaire asked for the following information: 1) pregnancy experience, 2) age at survey, maternal age, and ability to walk at pregnancy, 3) complications during pregnancy, 4) outcomes of pregnancy, 5) complications during delivery, 6) newborn complications, and 6) subjective disease progression during pregnancy and a year after delivery. In this study, disease onset was defined as the age when participants became aware of GNE myopathy symptoms, rather than the age at diagnosis or when test abnormalities were detected. Onset age and pathogenic variant data were collected from the Remudy database.

### Data analysis

Data are presented as mean ± standard deviation (SD), median, range, frequency, and percentage among respondents. We calculated 95% confidence intervals (CIs) for frequency data. Incidences of complications in post-onset pregnancies and deliveries were compared to those of the general Japanese population using Fisher’s exact test. *P* < 0.05 was considered statistically significant. With respect to post-onset pregnancies, slight significant differences were particularly difficult to detect due to the small sample size. To increase the sensitivity of our analysis, we also compared frequencies in all pregnancies (i.e., pre- and post-onset pregnancies) among patients with GNE myopathy to those of the general Japanese population. Since the development of muscle weakness/atrophy usually begins before patients notice any change, complications in pre-onset pregnancies, which are noted in average 4 years before patients become aware of any symptoms, might also be characteristics of GNE myopathy. All statistical analyses were performed using EZR on R 3.5.2 and R commander 2.5–1.

## Results

### Baseline characteristics

**Fig. 1:**
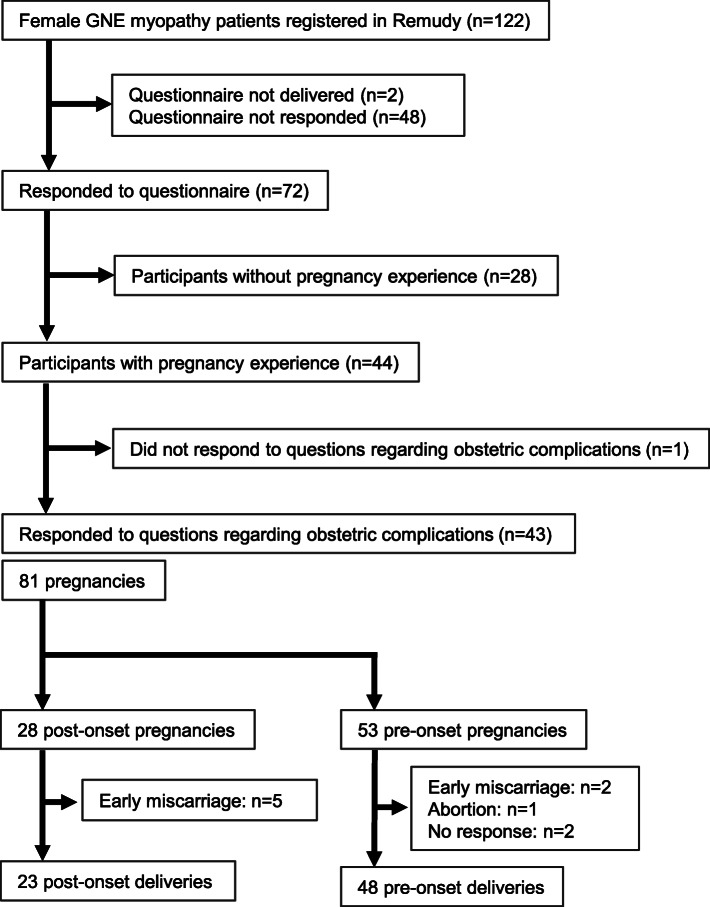
Participant recruitment. Questionnaires were sent to 122 Japanese female GNE myopathy patients registered in a national registry for neuromuscular diseases in Japan (Remudy)

Of the 120 female GNE myopathy patients who received the questionnaire, 72 respondents (60.0%) who answered questions relating to pregnancy experience were considered participants of this study (Fig. 1). Mean participant age was 51.0 ± 11.1 years (median, 49.5 years; range, 32–76 years) and mean onset age was 33.0 ± 9.8 years (median, 30 years; range, 16–62 years). There were no significant differences in distribution of participant age or onset age among respondents and non-respondents (data not shown). All 72 respondents were diagnosed genetically with biallelic pathogenic variants in *GNE*. Of the 72, 44 (61.1%) had pregnancy experience with an average of 1.8 pregnancies per person and a mean maternal age of 29.1 ± 5.0 years (median, 29 years; range, 19–40 years). Among 81 pregnancies reported, 28 (34.6%) were defined as post-onset pregnancies (i.e., those occurring after the onset of GNE myopathy). With regard to walking ability at pregnancy, 23/27 (85.2%) were able to walk without assistance, 4/27 (14.8%) used canes or assistive devices for walking, and none were unable to walk (27/28 post-onset pregnancies analyzed due to missing data) (Table 1).

**Table 1:** Participant background characteristics

Age				**Average [SD]**	**Median [Min-Max]**
	Age at survey (yrs)	Participants (*n* = 44)		51.0 [11.1]	49.5 [32-76]
	Onset age (yrs)^a^			33.0 [9.8]	30 [16-62]
	Maternal age (yrs)	Post-onset pregnancies (*n* = 28)		32.5 [4.4]	31.5 [23-40]
		Pre- and post-onset pregnancies (*n* = 81)		29.1 [5.0]	29.0 [19-40]
Genetic diagnosis				**Frequency** **% [95% CI]**	
	Homozygotes^a^	Participants (*n* = 44)		8.9 [2.5-21.2]	
	Compound heterozygotes^a^			91.1 [78.8-97.5]	
	c.620A>T (p.D207V) ^a^	Alleles (*n* = 88)		35.2 [25.3-46.1]	
	c.1807G>C (p.V603L) ^a^			30.7 [21.3-41.4]	
Parity			**n**		**n**
	0	Post-onset pregnancies (*n* = 28)	13	Pre- and post-onset pregnancies (n=81)	48
	1		11		29
	2 or more		4		4
Walking ability at pregnancy				**Frequency** **% [95%CI ]**	
	No assistance	Post-onset pregnancies (*n* = 27^b^)		85.1 [66.2-95.8]	
	Using assistive devices			14.8 [4.2-33.7]	
	Unable to walk			0.0 [0.0-12.8]	

^a^Data from the Remudy database. Other data are from the questionnaire

^b^Analyzed 27/28 post-onset pregnancies due to missing data (no response)

### Mode of conception

We asked participants about the mode of conception. Of 28 post-onset pregnancies, 26 (92.9[76.5–99.1]%) were achieved by spontaneous conception and 2 (7.1[0.9–23.5]%) by intrauterine insemination. None of the participants became pregnant using ovulation induction or by in vitro fertilization. The prevalence of spontaneous conception was similar to that of the general Japanese population in a previous cohort study (91.1%, *p* = 1) [17].

### Complications during pregnancy

We asked participants to provide information on obstetrical complications, such as hyperemesis gravidarum, threatened abortion, threatened preterm labor, hypertensive disorder of pregnancy, preeclampsia, gestational diabetes mellitus, abruptio placentae, placenta previa, blood type incompatibility, and others. The incidence of threatened abortion was 26.9% (7/26) in post-onset pregnancies, which was significantly higher than that of the general Japanese population (11.9%, *p* = 0.03) (26/28 pregnancies analyzed due to missing data) (Table 2). When 7 participants with post-onset pregnancies were asked whether they had ever been diagnosed with thrombocytopenia, 1 responded that she had a past history of thrombocytopenia. Among all pregnancies (i.e., pre- and post-onset pregnancies), threatened abortion occurred in 19.5% (15/77 pregnancies among 12 participants) (Supplementary Table 1) (77/81 pregnancies analyzed due to missing data). Participants with threatened abortion included 2 with a past history of thrombocytopenia.

**Table 2:** Complications during pregnancy in post-onset pregnancies among GNE myopathy patients

Complications	General population (Japanese)	Post-onset pregnancies (*n* = 26^a^)	Comparison
	Frequency %	Frequency % [95% CI]	***P*** value
Hyperemesis gravidarum		88.5 [69.8-97.6]	
Threatened abortion	11.9 [18]	26.9 [11.6-47.8]	0.03*
Threatened premature delivery	19.2 [18]	19.2 [6.6-39.3]	1.00
Hypertensive disorder of pregnancy	3.3 [18]	0.0 [0.0-13.2]	1.00
Preeclampsia		0.0 [0.0-13.2]	
Gestational diabetes mellitus	2.7 [18]	3.8 [0.1-19.6]	0.51
Abruptio placentae	0.5 [19]	0.0 [0.0-13.2]	1.00
Placenta previa	0.7 [19]	3.8 [0.1-19.6]	0.23
Blood type incompatibility		0.0 [0.0-13.2]	
Others		3.8 [0.1-19.6]	
		(prolapsed uterus)	

^a^Analyzed 26/28 pregnancies due to missing data (no response)

* ***p*** **< 0.05**

### Outcome of pregnancy

Incidences of early miscarriage, induced abortion, and delivery were 17.9% (5/28), 0.0% (0/28), and 82.1% (23/28), respectively, in post-onset pregnancies. Incidences of preterm delivery (defined as delivery before 36w6d), term delivery (delivery between 37w0d to 41w6d), and post-term delivery (delivery after 42w0d) were 8.7% (2/23), 91.3% (21/23), and 0.0% (0/23), respectively, in post-onset pregnancies. There were no late miscarriages or stillbirths. These incidences were not significantly different from those of the general population (Table 3). There were also no significant changes in prevalence of miscarriages, stillbirth, and term delivery in pre- and post-onset pregnancies compared with the general population (Supplemental Table 2).

**Table 3:** Outcomes of pregnancy in post-onset pregnancies among GNE myopathy patients

Outcomes	General population (Japanese)	Post-onset pregnancies (*n* = 28)	Comparison
	Frequency %	Frequency % [95% CI]	***P*** value
Early miscarriage	16-18 [20]^a^	17.9 [6.0-36.9]	
Late miscarriage		0.0 [0.0-12.3]	
Abortion		0.0 [0.0-12.3]	
Stillbirth	0.3 [19]	0.0 [0.0-12.3]	1.00
Delivery		82.1 [63.1-93.9]	
Preterm^b^	6.9 [19]	8.7 [1.1-28.0]	0.67
Term^b^	92.9 [19]	91.3 [72.0-98.9]	0.68
Post-term^b^	0.2 [19]	0.0 [0.0-14.8]	1.00

^a^Prevalence (%) of miscarriages, including both early and late miscarriages at maternal age up to 31 years old, increases with age

^b^Percentage among post-onset deliveries

### Complications during delivery

The majority of participants had vaginal deliveries; 82.6% (19/23) in post-onset deliveries. Incidences of emergency and elective cesarean section (CS) were 4.3% (1/23) and 13.0% (3/23), respectively, in post-onset deliveries. These incidences were not significantly different from those of the general population (Table 4). However, the prevalence of CS, especially emergency CS, was significantly lower in deliveries among GNE myopathy patients, including pre-onset deliveries (Supplementary Table 3).

**Table 4:** Complications during delivery in post-onset deliveries among GNE myopathy patients

		General population (Japanese)	Post-onset deliveries (*n* = 23)	Comparison
		Frequency %	Frequency % [95% CI]	***P*** value
Type of delivery	Vaginal delivery	81.9 [18]	82.6 [61.2-95.0]	1
	Cesarean section (CS)	18.1 [18]	17.4 [5.0-38.8]	1
	Emergency CS	13.7^a^	4.3 [0.1-21.9]	0.15
	Previous CS	8.1 [18]	8.7 [1.1-28.0]	0.71
	Breech presentation	2.4 [21]	4.3 [0.1-21.9]	0.43
Beginning of vaginal delivery^b^	Spontaneous labor		66.7 [41.0-87.7]	
	PROM	10-20 [22]^c^	16.7 [3.6-41.4]	
	Bleeding		0.0 [0.0-18.5]	
	Induced labor		16.7 [3.6-41.4]	
	Fetal abnormality		0.0 [0.0-18.5]	
	Maternal abnormality		0.0 [0.0-18.5]	
	Overdue pregnancy		5.6 [0.1-27.3]	
	Scheduled		11.1 [1.4-34.7]	
Outcomes of vaginal delivery^d,e^	No assistance		78.9 [54.4-93.9]	
	Vacuum extraction	7.6 [18]	10.5 [1.3-33.1]	0.39
	Manual fundal pressure	11.2 [23]	15.8 [3.4-39.6]	0.46
	Forceps delivery	0.3 [18]	0.0 [0.0-17.6]	
Management	Labor augmentation^f^		33.3 [13.3-59.0]	
	Blood transfusion	0.5 [17]	0.0 [0.0-14.8]	1
Number of fetuses	Singletons	98.1 [17]	100.0 [85.1-100.0]	1
Complications after delivery	Maternity blues		4.3 [0.1-21.9]	
	Postnatal depression		0.0 [0.0-14.8]	
	Deep vein thrombosis		0.0 [0.0-14.8]	
	Intrauterine infection		0.0 [0.0-14.8]	
	Mastitis		0.0 [0.0-14.8]	
	Prolapsed uterus		0.0 [0.0-14.8]	

*PROM* Premature rupture of the membrane

^a^Analyzed 3109 deliveries at Tokyo Medical and Dental University from 2013-2019

^b^Analyzed 18/19 post-onset vaginal deliveries due to missing data (no response)

^c^Frequency among deliveries

^d^Analyzed 19/19 post-onset vaginal deliveries

^e^Multiple choices allowed

^f^Analyzed 18/23 post-onset deliveries due to missing data (no response)

* ***p*** **<0.05**

Normally, labor begins spontaneously and is followed by spontaneous rupture of the membranes. The majority of deliveries began spontaneously by labor, i.e., 66.7% (12/18) in post-onset deliveries, while 16.7% (3/18) began with premature rupture of the membranes (PROM) and 16.7% (3/18) began with labor induction due to overdue pregnancy or scheduled delivery (18/23 deliveries analyzed due to missing data). Vacuum extraction and manual fundal pressure were employed due to prolongation of the second stage of labor in 10.5% (2/19) and 15.8% (3/19) of post-onset deliveries, respectively. These rates were not significantly different from those of the general Japanese population (19/23 deliveries analyzed due to missing data). The questionnaire included questions regarding management (labor augmentation and blood transfusion), number of fetuses, and complications after delivery (maternity blues, postnatal depression, deep vein thrombosis, intrauterine infection, mastitis, and prolapsed uterus). There were no significant differences in the incidences of these complications in post-onset deliveries among GNE myopathy patients compared to those of the general population (Table 4).

### Newborn outcomes

The mean body weight and height of newborns were 2962.0 ± 328.6 g (*n* = 26) and 48.8 ± 1.7 cm (*n* = 25), respectively, in post-onset deliveries. There were no cases of neonatal asphyxia, respiratory disorder, cardiac problems, or seizures (Table 5). Two newborns from the same pregnant mother who delivered before disease onset had intracranial hemorrhage (Supplementary Table 4).

**Table 5:** Newborn outcomes in post-onset deliveries among GNE myopathy patients

		General population (Japanese)	Post-onset deliveries (*n* = 23)		Comparison
Characteristics at birth		**Average**	**Average [SD]**	**Median [Min-Max]**	
	Body weight [kg]	2987 [447] [18]	2987.2 [339.9]	2940 [2218-3800]	
	Height [cm]	B: 49.0, G: 48.5 [24]	48.9 [1.7]	48.5 [45.5-52.0]	
		**Frequency %**	**Frequency** **% [95% CI]**		***P*** **value**
	Low birth weight (<2500 g)	9.4 [18]	4.3 [0.1-21.9]		0.72
Complications^a^			**Frequency** **% [95% CI]**		
	No complications		95.7 [78.1-99.9]		
	Prolonged jaundice		4.3 [0.1-21.9]		
	Others		0.0 [0.0-14.8]		

*B* boy, *G* girl

^a^Multiple choices allowed

### Symptoms of GNE myopathy during pregnancy and after delivery

We also asked how participants felt about their disease progression during pregnancy and after delivery compared to before pregnancy. Among participants with post-onset pregnancies, 10.5% (2/19, 95%CI: 1.3–33.1%) considered disease progression to be faster after pregnancy; these participants experienced difficulty in walking at an accelerated pace during the third trimester but required no assistive devices. On the other hand, 84.2% (16/19, 95%CI: 60.4–96.6%) considered it to be the same, and 5.2% (1/19, 95%CI: 0.1–26.0%) considered it to be slower (19/23 deliveries analyzed due to missing data). None of the participants developed myopathy during pregnancy. Of deliveries after disease onset, 19.0% (4/21, 95%CI: 5.4–41.9) considered disease progression a year after delivery to be faster compared to disease progression before pregnancy, whereas 80.9% (17/21, 95%CI: 58.1–94.6%) felt no difference (19/21 deliveries analyzed due to missing data). Among the former group, all felt difficulty in walking at an accelerated pace, 1 started requiring the use of an assistive device, 1 lost the ability to run, and 1 complained of exacerbated finger muscle weakness. Six participants developed myopathy within a year after delivery. The characteristics of these 6 participants are summarized in Table 6.

**Table 6:** Background of participants who experienced disease onset within a year after delivery

Age	Pathogenic variants		M	P	First symptoms	Feeding
23	c.620A > T (p.D207V)	c.1807G > C (p.V603L)	1	1	Frequent stumbling Difficulty in walking	B
26	c.1355 T > C (p.V452A)	c.1807G > C (p.V603L)	1	2	Difficulty in walking Unable to run	B
28	c.1355 T > C (p.V452A)	c.1998 T > A/G (p.N666K)	1	1	Finger muscle weakness	F
30	c.395G > A (p.R132H)	c.1807G > C (p.V603L)	NA	1	Frequent stumbling Difficulty in walking Unable to run	M
35	c.620A > T (p.D207V)	c.1807G > C (p.V603L)	NA	2	Frequent stumbling Difficulty in walking Difficulty in bending fingers	M
36	c.620A > T (p.D207V)	c.1807G > C (p.V603L)	3	1	Difficulty in walking Unable to run	B

*Age* Maternal age at pregnancy, *M* Months after delivery to disease onset, *P* Parity, *NA* data not available, *B* Breast feeding, *F* Formula feeding, *M* Mixed feeding

Participants were also asked about the method of feeding to determine whether sialic acid loss might be a risk factor for disease onset and/or exacerbation. Among 9 breastfeeding participants and 12 formula or mixed-feeding participants, 22.2% (2/9, 95%CI: 2.8–60.0%) and 16.7% (2/12, 95%CI: 2.1–48.4%), respectively, felt that their symptoms were exacerbated, while the remaining (7/9 and 10/12, respectively) participants felt no change. We also compared the type of feeding among participants who delivered before disease onset. Of 19 breastfeeding participants and 26 formula or mixed-feeding participants, 21.1% (4/19, 95%CI: 6.0–45.6%) and 7.7% (2/26, 95%CI: 0.9–25.1%), respectively, developed myopathy within a year after delivery.

## Discussion

Compared to results from a nationwide Japanese birth cohort study [18], we found no differences in frequency of natural conception, term delivery, or complications of newborns among pregnant GNE myopathy patients. However, the prevalence of threatened abortion was higher in post-onset pregnancies among GNE myopathy patients compared to the general population. Notably, the prevalence of CS, especially emergency CS, was lower in all pregnancies (i.e., pre- and post-onset deliveries) among GNE myopathy patients compared to the general population.

Threatened abortion is defined as pregnancy-related bloody vaginal discharge that occurs during the first half of a pregnancy and is accompanied by abdominal pain which may present as intermittent cramps [25]. This complication was noted relatively frequently among pregnancies of GNE myopathy patients, especially in post-onset pregnancies, compared to the general Japanese population. Maternal factors such as age, diabetes, thyroid disease, obesity, alcohol use, tobacco use, and illicit drug use are reported to increase the risk of threatened abortion [25]. No differences in age and prevalence of diabetes were found between pregnant GNE myopathy patients and women of the Japanese nationwide cohort study. Theoretically, vaginal bleeding may occur in pregnant women who have a tendency to bleed. Some case reports have indicated a relationship between thrombocytopenia and GNE myopathy [26] or GNE pathogenic variants [27]. Platelet desialylation level is reportedly associated with thrombocytopenia in septic patients [28]. Based on these findings, we analyzed responses regarding thrombocytopenia and low platelet counts from 12 participants who had threatened abortion. Two of the participants had a past history of thrombocytopenia and a bleeding tendency, which may have been the cause of threatened abortion. While a high prevalence of thrombocytopenia could be related to a high frequency of threatened abortion in GNE myopathy patients, this remains speculative and a better understanding of the prevalence and risks of threatened abortion is needed. Threatened abortion is associated with preterm labor, low birth weight, preeclampsia, preterm PROM, placental abruption, and intrauterine growth restriction [29]. However, neither the prevalence of threatened premature delivery nor the incidence of complications was high among GNE myopathy patients with threatened abortion in this study, possibly due to appropriate management.

The frequency of CS, especially emergency CS, was lower in all pregnancies (i.e., pre- and post-onset deliveries) among GNE myopathy patients compared to the general Japanese population. While over-rigidity of soft tissues in the lower birth canal is considered one of the causes leading to emergency CS [30], this may not be the case in patients with muscle weakness. The low frequency of CS may reflect the characteristics of pregnancies among GNE myopathy patients, although no significant difference was observed among those with post-onset deliveries compared to the general population, likely due to the small sample size. Conversely, the frequency of CS before the 37th gestational week is reportedly higher in patients with spinal muscular atrophy due to reduced lung function, and in some patients, pregnancy cannot be completed to term [31]. Most of our patients became pregnant pre-onset or in the early stage of GNE myopathy, and none lost ambulation at pregnancy. The prevalence of CS might vary in patients with advanced-stage GNE myopathy who have reduced respiratory function.

The impact of pregnancy or delivery on disease progression is a matter of great significance. A total of 7 patients were previously reported to have developed GNE myopathy during pregnancy [11–13]. Interestingly, in the present survey, none of the patients reported experiencing the first symptoms of GNE myopathy during pregnancy, while 6 did within a year after delivery. Among pregnancies after disease onset, roughly one-fifth of participants felt their disease progression accelerated after delivery, which was more frequent than those who felt their disease progression accelerated during pregnancy. This may suggest a tendency to develop GNE myopathy or accelerate disease progression within a year after delivery. We hypothesized that loss of sialic acid due to breastfeeding might be a risk factor, but no difference was found in subjective disease progression in breastfeeding participants compared to formula or mixed feeding participants. Breastfeeding participants had a higher frequency of developing disease within a year after delivery; however, the analyzed sample size was too small to draw conclusions. Reportedly, mean serum sialic acid concentration is significantly higher during pregnancy and decreases after delivery in healthy individuals [32]. A rapid decline in sialic acid concentration after delivery might trigger disease progression. In women with carnitine deficiency syndrome, carnitine supplementation is recommended during pregnancy as carnitine concentrations markedly decrease during gestation [33]. Sialic acid supplementation might help prevent deterioration of the disease due to pregnancy. However, sialic acid concentrations during pregnancy and postpartum need to be carefully monitored, as the level of sialic acid at 12 weeks postpartum is reportedly still higher than that of non-pregnant females [32], and since it remains unclear how different modes of feeding affect serum sialic acid concentrations. As for the influence of pregnancy on the disease course of other neuromuscular disorders, roughly half of patients with limb-girdle muscular dystrophy, one-third of those with spinal muscular atrophy, and one fifth of those with Charcot-Marie-Tooth disease reported deterioration of symptoms during pregnancy [31]. In another report, one-third of patients with myasthenia gravis (MG) experienced worsening of symptoms in the first trimester or postpartum, and in 15% of cases, pregnancy preceded the onset of MG. This may be explained by changes in the immune system and/or a decrease inα-fetoprotein following delivery, as well as stress and sleep deprivation [34]. The disease course of neuromuscular disorders including GNE myopathy is highly variable and unpredictable, making it difficult to examine the correlation with pregnancy. Moreover, other factors may affect the onset or subjective progression of symptoms, such as an increase in housework and childrearing, which might lead to a higher awareness of muscle weakness, as well as the lack of rest and sleep, which could accelerate disease progression. Monitoring of biomarkers or frequent scoring of muscle weakness will be needed to acquire more information.

This study has some limitations. First, the sample size was small, and thus the range of the 95% CI was wide and hindered the detection of significant differences. Nonetheless, it is difficult to obtain a large sample of patients with this very rare disease, and the present study represents one of the largest surveys of pregnancy in GNE myopathy patients. Second, we analyzed self-reported data. Self-reported data are not objective, and given that the mean respondent age was 17 years older than the mean maternal age, participants could have forgotten or had difficulty recalling their conditions during pregnancy. However, a previous study examined the reliability of retrospective self-reports on the prevalence of maternal morbidities, and found that data from these self-reports were consistent with clinical case notes [35]. Moreover, almost all parents in Japan use the Mother and Child Health Handbook (MCHH), an important record book of maternal and child health shared by parents and health providers at each checkup. Given its wide use [36], we assume that participant responses were mostly based on the MCHH, which is also reflected by the high response rate of birth weight and height. Third, there may have been selection bias, since those with severe phenotypes may have been more prone to participate in the national registry. Notwithstanding these potential limitations, we believe that this first and largest survey of pregnancy in Japanese GNE myopathy patients would be helpful for future patients who desire to get pregnant and for doctors responsible for managing their pregnancies.

## Conclusion

No serious complications were frequently observed among pregnant GNE myopathy patients. Moreover, the majority of the patients did not feel any changes in disease progression during pregnancy and after delivery. Our findings may give hope to patients who wish to get pregnant in the future. Meanwhile, the frequency of threatened abortion was high among pregnant GNE myopathy patients, and 6 patients developed the disease within a year after delivery. For better management of pregnancy among GNE myopathy patients, obstetricians should be mindful of the risk of threatened abortion, and neurologists should carefully monitor disease progression not only during pregnancy but also after delivery to ensure early support if and when symptoms arise or worsen.

## Supplementary information

**Additional file 1.**

**Additional file 2.**

## Data Availability

The datasets used and/or analyzed during the current study are available from the corresponding author, Madoka Mori-Yoshimura, on reasonable request.

## Abbreviations

CI: Confidence interval
CS: Cesarean section
ManNAc: UDP-N-acetylglucosamine 2-epimerase/N-acetylmannosamine
MG: Myasthenia gravis
NCNP: National Center of Neurology and Psychiatry
PADM: Patient association of distal myopathies
PROM: Premature rupture of the membranes
Remudy: National registry for neuromuscular diseases in Japan
SD: Standard deviation
UDP-GlcNAc: UDP-N-acetylglucosamine
