# Supplementary material for: Pregnancy in GNE myopathy patients: a nationwide repository survey in Japan
Source: Orphanet J Rare Dis. 2020 Sep 11;15:245. doi: 10.1186/s13023-020-01487-5 (PMC7488253; doi:10.1186/s13023-020-01487-5)
Supplement: Supplementary file 1 — Additional file 1. [file 13023_2020_1487_MOESM1_ESM.pdf]

Supplementary table 1. Complications during pregnancy in pregnancies among GNE myopathy patients including pre-onset pregnancies

Complications	General population (Japanese) Frequency %	Pre- and post-onset pregnancies (n=77[1)]) Frequency % [95% CI]	Comparison P value
**Hyperemesis gravidarum**		84.4 [74.6-91.7]	
**Threatened abortion**	11.9[18]	19.5 [11.3-30.1]	0.05
**Threatened premature delivery**	19.2[18]	10.4 [4.6-19.4]	0.06
**Hypertensive disorder of pregnancy**	3.3[18]	1.3 [0.03-7.0]	0.73
**Preeclampsia**		1.3 [0.03-7.0]	
**Gestational diabetes mellitus**	2.7[18]	1.3 [0.03-7.0]	0.73
**Abruptio placentae**	0.5 [19]	0.0 [0.0-4.7]	1.0
**Placenta previa**	0.7[19]	1.3 [0.03-7.0]	0.37
**Blood type incompatibility**		0.0 [0.0-4.7]	
**Others**		2.6 [0.3-9.1] (edema, prolapsed uterus)	

1) Analyzed 77/81 pregnancies due to missing data (no response).

Supplementary table 2. Outcomes of pregnancy in pregnancies among GNE myopathy patients including pre-onset pregnancies

Outcomes	General population (Japanese) Frequency %	Pre- and post-onset pregnancies (n=79[1)]) Frequency % [95% CI]	Comparison P value
**Early miscarriage**	16-18[20][2)]	8.9 [3.6-17.4]	
**Late miscarriage**		0.0 [0.0-4.6]	
**Abortion**		1.3 [0.03-6.9]	
**Stillbirth**	0.3[19]	0.0 [0.0-4.6]	1.0
**Delivery**		89.9 [81.0-95.5]	
**Preterm[3)]**	6.9[19]	2.5 [0.3-8.8]	0.24
**Term[3)]**	92.9[19]	95.8 [88.1-99.1]	0.49
**Post-term[3)]**	0.2[19]	1.3 [0.03-6.9]	0.14

1) Analyzed 79/81 pregnancies due to missing data (no response).

2) Percentage of miscarriages, including both early and late miscarriages at maternal age up to 31 years old. Prevalence miscarriages increased with age.

3) Percentage among deliveries including pre-onset deliveries.

Supplementary table 3. Complications during delivery in deliveries among GNE myopathy patients including pre-onset deliveries

		General population (Japanese)<br>Frequency<br>%	Pre- and post-onset deliveries (n=71)[1)]<br>Frequency<br>% [95% CI]	Comparison<br>P value
**Type of delivery**	Vaginal delivery	81.9[18]	90.1 [80.7-95.9]	0.088
	Cesarean section (CS)	18.1[18]	8.5 [3.2-17.5]	0.016*
	Emergency CS	13.7[2)]	2.8 [0.3-9.8]<br>(fetal distress, prolonged labor)	0.004*
	Previous CS	8.1[18]	2.8 [0.3-9.8]	0.12
	Breech presentation	2.4[21]	4.2 [0.9-11.9]	0.25
**Beginning of vaginal delivery[3)]**	Spontaneous labor		69.5 [56.1-80.8]	
	PROM	10-20[22][4)]	11.9 [4.9-22.9]	
	Bleeding		3.4 [0.4-11.7]	
	Induced labor		15.3 [7.2-27.0]	
	Fetal abnormality		1.7 [0.04-9.1]	
	Maternal abnormality		3.4 [0.4-11.7]	
	Overdue pregnancy		6.8 [1.9-16.5]	
	Scheduled		3.4 [0.4-11.7]	
**Outcomes of vaginal delivery[5), 6)]**	No assistance		78.1 [66.0-87.5]	
	Vacuum extraction	7.6[18]	12.5 [5.6-23.2]	0.13
	Manual fundal pressure	11.2[23]	12.5 [5.6-23.2]	0.69
	Forceps delivery	0.3[18]	0.0 [0.0-5.6]	1
**Management**	Labor augmentation[7)]		30.5 [19.2-43.9]	
	Blood transfusion	0.5[17]	1.4 [0.04-7.6]	1
**Number of fetuses**	Singletons	98.1[17]	100.0 [95.1-100.0]	0.41
**Complications after delivery**	Maternity blues		2.7 [0.3-9.4]	
	Postnatal depression		0.0 [0.0-4.9]	

Deep vein thrombosis	0.0 [0.0-4.9]
Intrauterine infection	1.4 [0.0-7.3]
Mastitis	8.1 [3.0-16.8]
Prolapsed uterus	0.0 [0.0-4.9]

PROM: Premature rupture of the membrane.

1) Analyzed 71/73 deliveries due to missing data (no response).

2) Analyzed 3109 deliveries at Tokyo Medical and Dental University from 2013-2019.

3) Analyzed 59/68 vaginal deliveries due to missing data (no response).

4) Frequency among vaginal deliveries and CS.

5) Analyzed 64/68 vaginal deliveries due to missing data (no response).

6) Multiple choices allowed.

7) Analyzed 59/73 deliveries due to missing data (no response).

***p<0.05**

Supplementary table 4. Newborn outcomes in deliveries among GNE myopathy patients including pre-onset deliveries

		General population (Japanese)	Pre- and post-onset deliveries (n=71)		Comparison
**Characteristics**		**Average**	**Average [SD]**	**Median [Min-Max]**	
**at birth**	Body weight [kg][1)]	2987 [447][18]	3003.6 [344.6]	2937 [2218-3800]	
	Height [cm][2)]	B: 49.0, G: 48.5[26]	49.2 [1.7]	49.0 [45.5-53.0]	
		**Frequency**	**Frequency**		**P value**
		**%**	**% [95% CI]**		
	Low birth weight (<2500 g)[1)]	9.1[18]	2.9 [0.4-10.2]		0.09
**Complications[3)4)]**			**Frequency**		
			**% [95% CI]**		
	No complications		75.4 [63.5-84.9]		
	Prolonged jaundice		4.4 [0.9-12.2]		
	Intracranial hemorrhage		2.9 [0.4-10.1]		
	Vomiting		1.4 [0.04-7.8]		
	Others		1.4 [0.04-7.8] (hypospadias, ptosis)		

B: boy, G: girl

1) Analyzed 68/71 newborns due to missing data (no response).

2) Analyzed 65/71 newborns due to missing data (no response).

3) Analyzed 69/71 newborns due to missing data (no response).

4) Multiple choices allowed.
