# Supplementary material for: Pregnancy in GNE myopathy patients: a nationwide repository survey in Japan
Source: Orphanet J Rare Dis. 2020 Sep 11;15:245. doi: 10.1186/s13023-020-01487-5 (PMC7488253; doi:10.1186/s13023-020-01487-5)
Supplement: Supplementary file 2 — Additional file 2. [file 13023_2020_1487_MOESM2_ESM.pdf]

A : Questions about pregnancy

＊If you have 2 or more pregnancies, please take a copy.

＊ Technical terms are described in original Japanese version.

A-1

	pregnancy
① Age at survey	years old
Age at onset (the age you became aware of GNE myopathy symptoms, rather than the age at diagnosis or when test abnormalities were detected)	years old
Age at pregnancy	years old
② Number of fetus	
③ Walking ability at pregnancy	1☐ before onset<br>2☐ able to walk without assistive devices<br>3☐ able to walk with assistive devices<br>4☐ unable to walk
④ Need of assistance for normal daily activities such as eating meals, bathing, dressing, use of toilet, … etc.	1☐ do not need assistance for normal daily activities<br>2☐ need assistance for normal daily activities

A- 2

① How did you get pregnant？	1☐ natural conception<br>2☐ intrauterine insemination<br>3☐ ovulation induction<br>4☐ in-vitro fertilization
② ▼ If you have answered "natural conception", please answer the question below.	
・Did you planned to get pregnant?	1☐ yes<br>3☐ no<br>2☐ not planned, but was desiring to have a child someday

A- 3

① Did you have past history before getting pregnant？<br>・ If yes, please describe in detail.	1☐ no<br>2☐ yes<br>[ ]
・ Have you ever diagnosed as thrombocytopenia?	1☐ no<br>2☐ previously diagnosed<br>3☐ currently diagnosed

A-4

Did you have any complications during pregnancy? If yes, please describe in detail.	
① ▶hyperemesis gravidarum	1☐ no<br>2☐ yes, no treatment required<br>3☐ yes, outpatient treatment<br>4☐ yes, hospitalized（duration: ）<br>5☐ no idea
② ▶threatened miscarriage	1☐ no<br>2☐ yes（weeks of pregnancy: [ ] wks)<br>→ 21☐ abnormal bleeding 22☐ abdominal pain 23☐ no symptoms<br>24☐ outpatient treatment<br>25☐ hospitalized: duration [ ] wks<br>26☐ cervical cerclage
③ ▶pregnancy induced hypertension	1☐ no<br>2☐ yes（weeks of pregnancy: [ ] wks)<br>→ 21☐ rest at home<br>22☐ oral antihypertensive therapy without hospitalisation<br>23☐ hospitalisation with bed rest<br>24☐ hospitalisation with oral antihypertensive therapy<br>25☐ hospitalisation with other treatment<br>26☐ hospitalisation and delivered early (vaginal, cesarean section)

A-4

④ ▶Gestational diabetes mellitus	1☐ no<br>2☐ yes, medical nutrition therapy<br>3☐ yes, insulin therapy
⑤ ▶Seizures during pregnancy	1☐ yes 2☐ no
⑥ ▶Premature separation of normally implanted placenta	1☐ yes 2☐ no
⑦ ▶Placenta previa	1☐ yes 2☐ no
⑧ ▶Blood type incompatibility	1☐ no<br>2☐ yes<br>→ 21☐ cesarean section<br>22☐ exchange transfusion<br>23☐ fetal transfusion<br>24☐ plasma exchange<br>25☐ other ( )
⑨ ▶Threatened premature delivery	1☐ no<br>2☐ yes (weeks of pregnancy: [ ] wks)<br>→ 21☐ bleeding 22☐ rupture of the membrane 23☐ uterine contraction<br>24☐ no symptoms (shortening of the cervical canal etc)<br>25☐ rest at home, medication<br>26☐ hospitalized with medication: duration [ ] wks
⑩ ▶Others	[ ]
⑪ Result of pregnancy	1☐ early miscarriage<br>2☐ late miscarriage<br>3☐ abortion<br>→ 31☐ for social / economical reasons<br>32☐ for maternal body / health<br>33☐ for fetal problems (congenital malformation etc.)<br>34☐ other reasons [ ]<br>4☐ stillborn: getational age ( ) wks<br>**【End of the survey. Thank you.】**<br>5☐ delivered<br>↓ Please proceed to A-5"About delivery".<br>↓

A-5

▼ To who have answered "delivered" in A-4-②	
① Timing of delivery	1☐ term delivery (gestational age: 37 to 41 weeks)<br>2☐ preterm delivery (gestational age: less than 37 weeks)<br>3☐ post-term delivery (gestational age: more than 42 weeks)
	▼Please fill the timing of delivery if you know.<br>[ ] wks [ ] days
② Type of delivery you wished to have	1☐ wished to have vaginal delivery → proceed to ④<br>2☐ wished to have cesarean section → proceed to ③
**▼ To who have answered "wished to have cesarean section" in A-5-②**	
③ Why did you wished to have cesarean section?	1☐ bleech presentation<br>2☐ previous cesarean section<br>3☐ after uterus surgery<br>4☐ cephalopelvic disproportion<br>5☐ underdeveloped fetus<br>6☐ maternal problems<br>7☐ others:<br>[ ] → Proceed to ⑧
**▼ To who have answered "wished to have vaginal delivery" in A-5-②**	
④ What was the reason for hospitalisation?	1☐ spontaneous labor<br>2☐ rupture of the membrane<br>3☐ bleeding<br>4☐ overdue pregnancy<br>5☐ fetal abnormality<br>6☐ scheduled<br>7☐ other reason<br>[ ]
⑤ Did you have painless delivery ?	1☐ yes 2☐ no
⑥ Did you use labor-inducing drugs ?	1☐ yes 2☐ no<br>3☐ no idea

A-5

⑦ How was the delivery managed?	1☐ spontaneous vaginal delivery<br>2☐ manual fundal pressure<br>3☐ vacuum extraction<br>4☐ forceps delivery<br>5☐ cesarean section (reasons)<br>→ 51☐ prolonged labor<br>52☐ fetal distress<br>53☐ overdue pregnancy<br>54☐ multiple pregnancy<br>55☐ intrauterine infection<br>56☐ others<br>[ ]

▼ To all	
⑧ How was the amount of bleeding?	1☐ normal, not mentioned<br>2☐ massive bleeding, transfusion not required<br>3☐ massive bleeding, transfusion
⑨ Did you have any complications after delivery ?	1☐ no<br>2☐ yes<br>→ 21☐ maternity blues<br>22☐ deep vein thrombosis<br>23☐ intrauterine infection (treated with antibacterial drug)<br>24☐ mastitis<br>25☐ postnatal depression<br>26☐ descent of utereus<br>27☐ prolapsed uterus<br>28☐ other<br>[ ]
⑩ How many children have you delivered before?	1 ・ 2 ・ 3 ・ 4 ・ 5

A- 6

① How did you feel about the symptoms of GNE myopathy during pregnancy?	1☐ Was not aware of any symptoms until delivery.<br>2☐ Was not aware of any symptoms at pregnancy. First symptoms were during pregnancy. (weeks of pregnancy: [______]wks)<br>3☐ Pregnancy was after onset. Progression of the symptoms were same during pregnancy.<br>4☐ Pregnancy was after onset. Progression of the symptoms were slower during pregnancy.<br>5☐ Pregnancy was after onset. Progression of the symptoms accelerated during pregnancy. (Progression accerelated from [ ]wks of pregnancy.)<br>6☐ Others<br>[ ]<br>(The doctor prescribed complete rest, so hard to select. etc)
② How did you feel about the symptoms of GNE myopathy after delivery?	1☐ Was not aware of any symptoms at least a year after delivery.<br>2☐ Was not aware of any symptoms at delivery. First symptoms were within a year after delivery. (weeks after deliverey: [ ]mths)<br>3☐ Was not aware of any symptoms at delivery. First symptoms were during pregnancy, but disappeared after delivery.<br>4☐ Delivery was after onset. Progression of the symptoms got better after delivery, but not to the level before pregnancy.<br>5☐ Delivery was after onset. Progression of the symptoms accelerated after delivery. (Progression accerelated from [ ]mths to [ ]mths after delivery.)<br>6☐ Delivery was after onset. Progression of the symptoms were same after delivery.<br>7☐ Others<br>[ ]
③ If you had first symptoms of GNE myopathy during pregnancy or within a year after delivery, what was the first symptom?	1☐ difficulty in walking<br>→ usage of stics/wheelchair（11☐ yes ・12☐ no ）<br>2☐ frequent stumbling<br>3☐ unable to run<br>4☐ unable to climb stairs<br>5☐ difficulty in bending fingers<br>6☐ others<br>[ ]
④ If you felt progression of the disease accerelated during pregnancy or within a year after delivery, how did it change?	1☐ acceleration of difficulty in walking<br>2☐ unable to walk<br>3☐ started to use sticks / wheelchair<br>4☐ symptoms of fingers / arms (Please describe below.)<br>[ ]<br>5☐ others<br>[ ]

B : About newborn.

＊If newborn have 3 or more newborns, please take a copy.
＊Technical terms are described in original Japanese version.

B-1

① Newborn (after birth to 1-week-old)

	First child		Second child	
▶1 Birth weight	g		g	
▶2 Height at birth	c m		c m	
▶3 Apgar score	1 1min [ ] points		1 1min [ ] points	
	2 5min [ ] points		2 5min [ ] points	
▶4 Did newborn have abnormalities at birth?	1 ☐yes	2 ☐no	1 ☐yes	2 ☐no
▶5 Did newborn have fetal distress?	1 ☐yes	2 ☐no	1 ☐yes	2 ☐no
▶6 Did newborn have respiratory problems	1 ☐yes	2 ☐no	1 ☐yes	2 ☐no
▼ Who selected "yes", please check the applicable bo:				
・Meconium aspiration syndrome	11 ☐		11 ☐	
・Transient tachypynea of the newborn	12 ☐		12 ☐	
・Apnea	13 ☐		13 ☐	
・Others (Please describe the detail.)	14 [ ]		14 [ ]	
・Uncertain	15 ☐		15 ☐	
▶7 Did newborn have cardiovascular disease？	1 ☐yes	2 ☐no	1 ☐yes	2 ☐no
▼ Who selected "yes", please check the applicable bo:				
・Heart malformation	11 ☐		11 ☐	
・Persistent pulmonary hypertension of the newborn	12 ☐		12 ☐	
・Others (Please describe the detail.)	13 [ ]		13 [ ]	
▶8 Did newborn have jaundice？	1 ☐yes	2 ☐no	1 ☐yes	2 ☐no
▶9 Did newborn have a convulsion？	1 ☐yes	2 ☐no	1 ☐yes	2 ☐no
▶10 Did newborn have intracranial bleeding？	1 ☐yes	2 ☐no	1 ☐yes	2 ☐no
▶11 ・Others (Please describe the detail.)	[ ]		[ ]	

②About development

	First child		Second child	
▶1 Head holding		months		months
▶2 Start sitting		months		months
▶3 Start walking alone（without holding)	years	months	years	months

③ Diseases a child had. (1)

	First child		Second child	
▶1 Asthma	1 ☐yes	2 ☐no	1 ☐yes	2 ☐no
▶2 Atopic dermatitis	1 ☐yes	2 ☐no	1 ☐yes	2 ☐no
▶3 Allergy（food・dust・drug etc.)	1 ☐yes	2 ☐no	1 ☐yes	2 ☐no
・Who selected "yes", please check the applicable box.	11 [ ]		11 [ ]	

B-1

③ Diseases a child had. (2)

	First child	Second child
▶ Others.		
1 ・Please describe when and what the disease was.	Disease : ( ) years old	Disease : ( ) years old
2	Disease : ( ) years old	Disease : ( ) years old
3	Disease : ( ) years old	Disease : ( ) years old
4	Disease : ( ) years old	Disease : ( ) years old

④ About nutrition.

	First child	Second child
▶ Nutrition after birth.	1 ☐Breastbreeding 2 ☐Mixed breeding 3 ☐Formula breeding	1 ☐Breastbreeding 2 ☐Mixed breeding 3 ☐Formula breeding

Thank you for the corporation.
